# Thyroidectomy without lymph node dissection should be considered for stage T1 medullary thyroid carcinoma: a population-based cohort study

**DOI:** 10.3389/fendo.2024.1433329

**Published:** 2024-08-29

**Authors:** Zixia Tao, Xianzhao Deng, Zheng Ding, Bomin Guo, Youben Fan

**Affiliations:** Department of General Surgery, Thyroid and Parathyroid Center, Shanghai Sixth People’s Hospital Affiliated to Shanghai Jiao Tong University School of Medicine, Shanghai, China

**Keywords:** stage T1, medullary thyroid carcinoma, surgical procedures, lymph node dissection, SEER, propensity score matching (PSM)

## Abstract

**Background:**

The necessity and therapeutic value of lymph node dissection (LND) in early stage T1 MTC patients remain controversial.

**Methods:**

Patients with T1MTC were identified from the Surveillance, Epidemiology, and End Results (SEER) database. Poisson regression analysis was utilized to investigate promotive factors for lymph node metastasis in T1MTC patients. Fisher’s exact test was employed to calculate baseline differences between non-LND and LND groups. Propensity score match (PSM) was used to control baseline bias. Survival outcomes were calculated by Kaplan-Meier method and log-rank test. Multivariable Cox regression assessed the prognostic impact of LND across subgroups.

**Results:**

Of 3298 MTC cases, 50.4% were T1MTC. The lymph node metastasis rate increased along with the T stage (from 22.2% to 90.5%). Among 1231 T1MTC patients included after exclusion criteria, 72.0% underwent LND and 22.0% had lymph node metastasis. Patients aged younger than 44 years (RR=1.700, p<0.001), male (RR=1.832, p<0.001), and with tumor larger than 10mm (RR=2.361, p<0.001) were more likely to have lymph node metastasis, while elderly patients (p<0.001) and those with microcarcinoma (p<0.001) were more likely to undergo non-LND procedures. LND provided no OS or DSS benefit over non-LND before and after propensity score match (matched 10-year OS/DSS: LND 83.8/96.2% vs non-LND 81.9/99.3%, p>0.05). Subgroup analyses revealed no prognostic gain with LND in any subgroup (p>0.05).

**Conclusion:**

Nearly half of MTC patients were diagnosed at T1 stage and had low lymph node risk. Different from ATA guidelines, avoiding routine LND conferred similar prognosis to standard procedures while potentially improving quality of life. Large-scale prospective multi-center studies should be conducted to further validate these findings.

## Introduction

Medullary thyroid carcinoma (MTC) arises from thyroid parafollicular cells, characterized by specific secretion of calcitonin and carcinoembryonic antigen (CEA). Since endocrine suppressive therapy and iodine-131 therapy are ineffective, surgery remains the only curative option for MTC (1–3). The 2015 ATA guidelines recommended total thyroidectomy (TT) with bilateral central lymph node dissection (LND) with/without lateral LND for all MTC patients, considering potential multifocality and high nodal risk in hereditary cases (~25%) (1). However, this standard approach raises concerns over high complication rates and overtreatment for early-stage MTC (4–8).

Total thyroidectomy with bilateral LND increased the risk of harm bilateral recurrent larynx nerve and parathyroids. A recent multicenter cohort study revealed that the hypoparathyroidism rate and recurrent laryngeal nerve paralyzes rate in patients who underwent surgical procedures are 43.8-56% and 11.3-28%, respectively, which means that these patients may require regular oral or intravenous calcium supplements, lose their normal speech voice or even be unable to speak. Interestingly, the researchers found limiting the scope of LND reduced these to 29% and 6%, respectively (9). Another controversial issue is the overtreatment of standard surgical procedures in early-stage MTC patients. Patients with ≤2 cm intrathyroidal tumors were defined as stage T1 medullary thyroid carcinoma (T1MTC) (10). According to past studies, T1MTC constitutes approximately 43-68% of all MTC cases, and has a good prognosis (10-year DSS: 97%), even patients who develop regional metastases tend to survive for a long time (4, 5, 11, 12). Intriguingly, ATA guidelines allow for incomplete surgery in early-stage MTC without hereditary traits or elevated postoperative calcitonin, implying cure is possible with less than total thyroidectomy and standard lymph node dissection (1).

In light of these doubts about the practices recommended by the ATA guidelines, several recent studies have investigated the optimal surgical procedure for early-stage sporadic MTC patients and supported unilateral thyroidectomy as curative for selected early sporadic MTC (5, 6, 13). However, past studies have mainly focused on the extent of thyroidectomy, and data on optimal lymph node dissection and outcomes in early MTC are scarce. What’s more, clinical data on these studies have been primarily gathered from retrospective institutional experience. Large-scale population-based analyses on this topic are warranted to understand its full clinical implications.

In the present study, we utilized the SEER database to demonstrate the N-stage distribution at different T stages in MTC patients, for the first time. In addition, we compare survival outcomes between LND and non-LND groups in T1MTC patients by propensity score matching for the first time. Further subgroup analyses were also performed to verify the benefits of LND in different subgroups. The results of our study indicated that since the low lymph node metastasis risk in T1MTC patients, LND failed to improve the prognosis, indicating that avoiding LND in T1MTC patients is viable.

## Materials and methods

### Data sources

The data for patients with T1 stage MTC who were diagnosed between 2004 and 2020 were extracted using SEER*STAT software (v8.4.0, https://seer.cancr.gor/, published in April 2023) The Surveillance, Epidemiology, and End Results (SEER) program includes 17 high-quality population-based registries, containing 28% of the US population.

**Figure 1:**
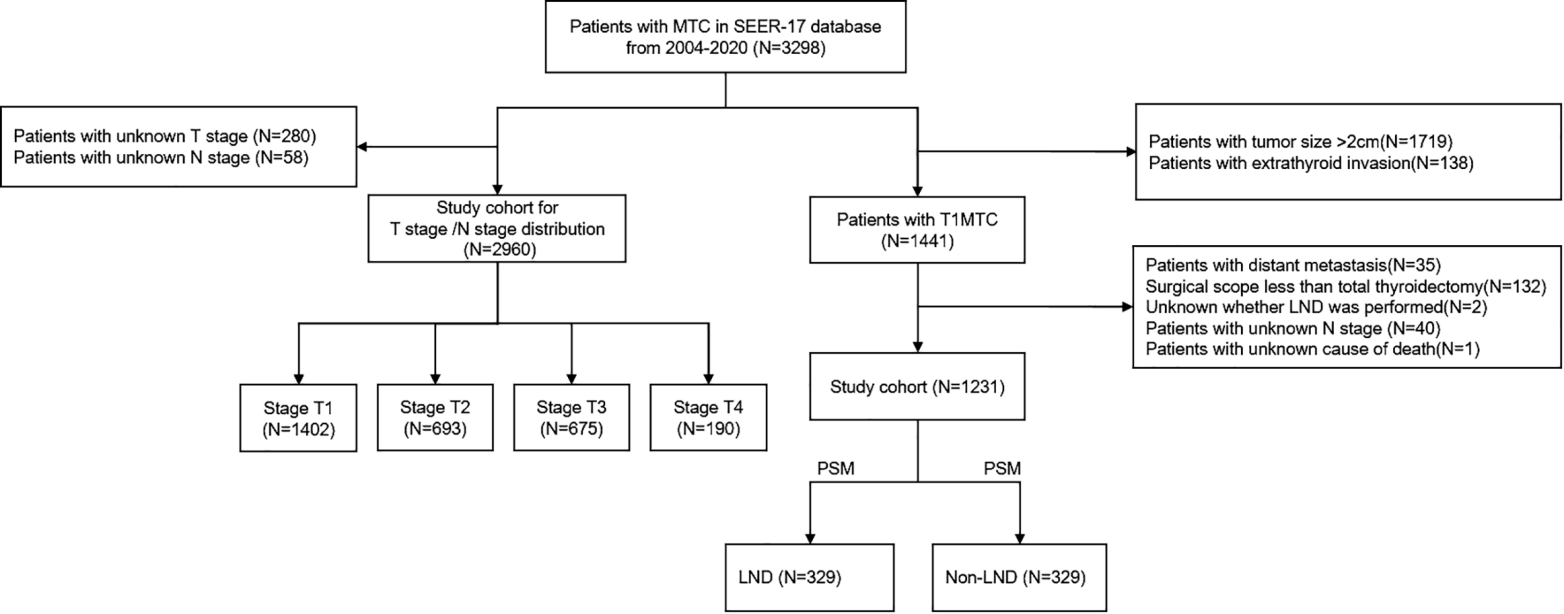
The inclusion, exclusion, and divided criteria of the SEER cohort. MTC, Medullary thyroid carcinoma; SEER, Surveillance, Epidemiology, and End Results database; T1MTC, T1 stage medullary thyroid carcinoma; PSM, Propensity score matching; LND, Lymph node dissection.

The study cohort was selected by the following variables. “Primary site label” and “ICD3-O-3” variables were set to C73.9 and 8845,8510-8513, respectively, for screening MTC patients. “Diagnostic Confirmation” and “Type of reporting source” variables were used to exclude patients confirmed by microscopy, autopsy, and death certificates. Demographic characteristics were extracted through “Sex”, “Age recode with single ages and 90+”, “Race recode (W, B, AI, API)”, and “Marital status at diagnosis” variables. Age was divided into categorical variables: 0-44, 45-64, ≥65 years, corresponding to young, middle-aged, and old patients, respectively. “Cs Tumor (2004-2015)”, “Derived SEER Combined (2016-2017)” and “EOD Primary tumor (2018+)” variables were used to extract clinicopathological characteristics. Treatment-related characteristics and outcome data were gained from “Rx Summ-Surg Prim site”, “Regional lymph nodes examined”, “Survival months”, “Vital status recode” and “SEER case-specific death classification” variables. The SEER program updates follow-up data annually, at minimum monthly intervals, and the follow-up end point was December 2020. Patients with tumor size ≤20mm and no extrathyroidal extension were included in the SEER cohort, while patients with surgical scope less than total thyroidectomy, unknown N stage, unknown lymph node dissection, and distant metastasis were excluded. **Figure 1** illustrates the detailed inclusion and exclusion process. The work has been reported in line with the Strengthening the Reporting of Observational Studies in Epidemiology (STROBE) statement (14).

### Statistical analyses

Categorical variables were expressed as percentages and continuous variables were expressed as mean± standard deviation or median with percentiles. Modified Poisson regression analysis was utilized to investigate promotive factors for lymph node metastasis in T1MTC patients. The Fisher’s exact test was used to assess baseline level differences between the non-LND group and the LND group. To reduce the influence of the selection bias, the PSM method of logistic regression analysis was used to match the clinicopathological features of the patients in these two groups with a matching ratio of 1:1, nearest neighbor caliper matching, with a caliper value of 0.2. Based on previous studies, the baseline level of four characteristics, namely, sex, age at diagnosis, marital status and tumor size were corrected by PSM. The Kaplan-Meier method and log-rank test were used to establish the survival curves of matched groups and compare the overall survival (OS) rates and disease-specific survival (DSS) rates. Multivariate Cox regression analysis was used for subgroup analyses of LND efficacy, with all results adjusted by age, sex, tumor size, race, and marital status. All tests were two-tailed with α defined as 0.05. The confidence interval (CI) was set at 95%. All the statistical analyses were performed by R software (V4.2.1) or IBM SPSS Statistics 25. The “Matchit” package was used for PSM and the “Mice” package was employed for multiple imputations.

## Results

### T stage and N stage distribution in the SEER database

**Figure 2:**
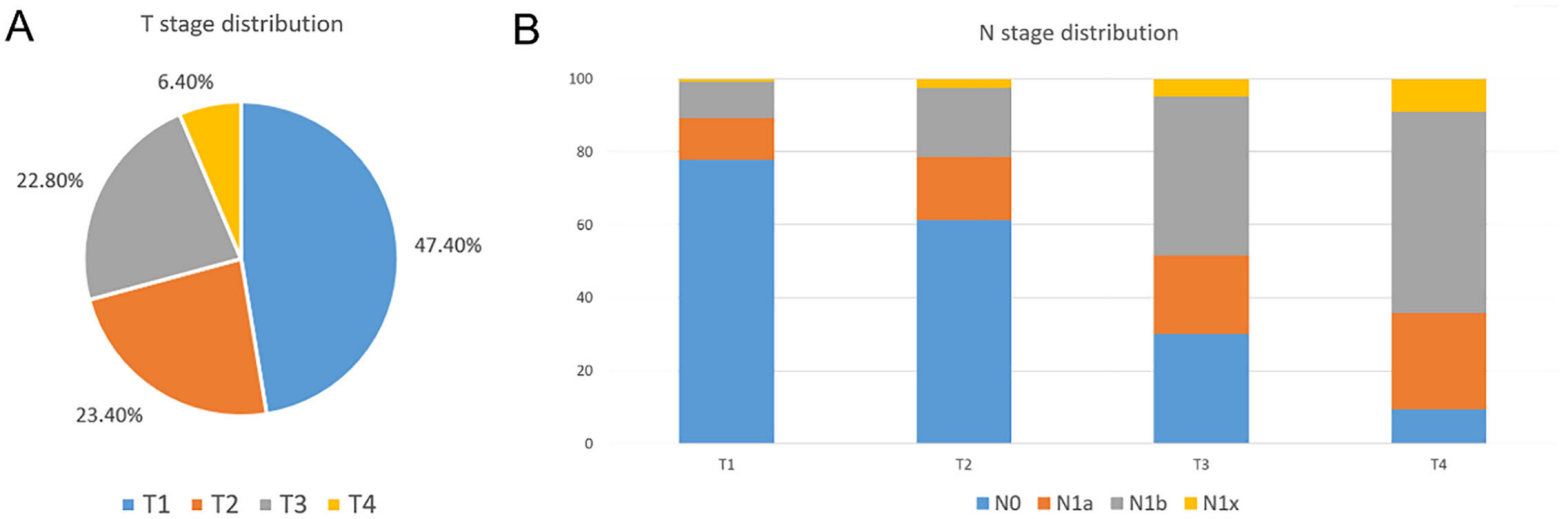
T stage distribution and N stage distribution at different T stage in the SEER database. **(A)** T stage distribution of MTC patients in the SEER database; **(B)** N stage distribution by T stage in the SEER database.

A total of 3298 MTC patients were identified from the SEER database between 2004 and 2020. **Figure 2** shows T stage distribution and N stage distribution by different T stages. The figure demonstrated that T1MTC patients accounted for approximately 47.4% of all MTC patients. Additionally, we found that the lymph node metastasis rate increased with higher T stage, and T1MTC patients had the lowest rate (22.2%), while rates for stage T2, T3, and T4 patients were 37.5%, 71.5%, and 90.5%, respectively.

### Clinicopathological features, and promotive factors for lymph node metastasis in T1MTC patients

A total of 1231 T1MTC patients were included in our study cohort from the SEER database. Among 1231 T1MTC patients, 65.6% were female and 41.6% were diagnosed at 45-64 years. Moreover, 51.8% had tumor size <10 mm. Additionally, LND was performed in approximately 72.0% of patients, but lymph node metastasis was present in only 22.0%. The mean follow-up period of the entire cohort was 81.0 months, with 123 deaths and 25 disease-specific deaths. Detailed clinicopathological characteristics are demonstrated in **Table 1**.

**Table 1:** Clinical characteristics of patients with stage T1 medullary thyroid carcinoma from 2004-2020 in the SEER cohort.

Characteristics	No. of patients (N)	Percentage (%)
Total	1231	100.0
Age at diagnosed, years/Sex		
0-44/M	121	9.8
0-44/F	241	19.6
45-64/M	175	14.2
45-64/F	338	27.4
≥65/M	127	10.4
≥65/F	229	18.6
Race (N=1215)		
White	1043	85.8
Black	94	7.7
Others	78	6.4
Marital status (N=1170)		
Married	687	58.7
Not married	483	41.3
Household income, $		
<60,000	934	75.9
≥60,000	297	24.1
Tumor size, mm		
1-10	638	51.8
11-20	593	48.2
N stage		
N0	960	78.0
N1a	150	12.2
N1b	121	9.8
LND		
No	345	28.0
Yes	886	72.0
Follow-up time, months(Mean ± SD)		
	81.0 ± 55.1	–
Survival status		
Death	123	10.0
Alive	1108	90.0
Disease-specific survival status		
Dead of MTC	25	2.0
Others	1206	98.0

F, Female; M, Male; LND, Lymph node dissection; SD, Standard deviation; MTC, Medullary thyroid carcinoma.

Besides, modified Poisson regression analysis demonstrated young age (RR=1.700, p<0.001), male (RR=1.832, p<0.001), and tumor larger than 10mm (RR=2.361, p<0.001) were significantly independent promotive factors for lymph node metastasis in T1MTC patients. (**Table 2**).

**Table 2:** Promotive factors for lymph node metastasis in T1MTC patients.

Characteristics	Multivariable analysis		
	RR	95% CI	P-value
Age at diagnosis, 0-44 years	Ref.		
Age at diagnosis, 45-64 years	0.772	0.610-0.978	0.032
Age at diagnosis, ≥65 years	0.588	0.446-0.775	<0.001
Sex, Female	Ref.		
Sex, Male	1.823	1.490-2.230	<0.001
Race, White	Ref.		
Race, Black	1.033	0.712-1.450	0.863
Race, others	0.712	0.444-1.144	0.161
Tumor size, 1-10mm	Ref.		
Tumor size, 11-20mm	2.361	1.877-2.970	<0.001

RR, Relative risk; CI, Confidence interval.

### Baseline level, outcomes, adjusted outcomes in the LND and non-LND groups

The SEER cohort was divided into LND and non-LND groups based on whether LND was performed. **Table 3** illustrates the baseline characteristics of the two groups before and after PSM. Before PSM, there were significant differences in age (P<0.001) and tumor size (P<0.001) between the two groups. Namely, the non-LND group had a higher proportion of elderly patients (36.8%) and microcarcinoma (60.0%), compared to the LND group (25.9%, 48.6%). These differences were corrected by PSM, and all the baseline levels of these two groups achieved consistent.

**Table 3:** Baseline difference in patients with stage T1 medullary thyroid carcinoma before and after PSM in the SEER cohort.

	Before PSM, N (%)			After PSM, N (%)		
Characteristics	LND	Non-LND	P-value	LND	Non-LND	P-value
Total	886 (100.0)	345 (100.0)	–	329 (100.0)	329 (100.0)	–
Sex			0.095			1.000
Male	317 (35.8)	106 (30.7)		105 (31.9)	105 (31.9)	
Female	569 (64.2)	239 (69.3)		224 (68.1)	224 (68.1)	
Age at diagnosed, years			<0.001			1.000
0-44	282 (31.8)	80 (23.2)		80 (24.3)	80 (24.3)	
45-64	375 (42.3)	138 (40.0)		130 (39.5)	130 (39.5)	
≥65	229 (25.9)	127 (36.8)		119 (36.2)	119 (36.2)	
Marital status			0.652			1.000
Married	524 (59.1)	199 (57.7)		194 (59.0)	194 (59.0)	
Not married	362 (40.9)	146 (42.3)		135 (41.0)	135 (41.0)	
Tumor size, mm			<0.001			1.000
1-10	431 (48.6)	207 (60.0)		195 (59.3)	195 (59.3)	
11-20	455 (51.4)	138 (40.0)		134 (40.7)	134 (40.7)	
N stage			–			–
N0	615 (69.4)	345 (100.0)		243 (73.9)	329 (100.0)	
N1a	150 (16.9)	0 (0.0)		42 (12.8)	0 (0.0)	
N1b	121 (13.7)	0 (0.0)		44 (13.3)	0 (0.0)	

PSM, Propensity score matching; LND, Lymph node dissection; MTC, Medullary thyroid carcinoma.

**Figure 3:**
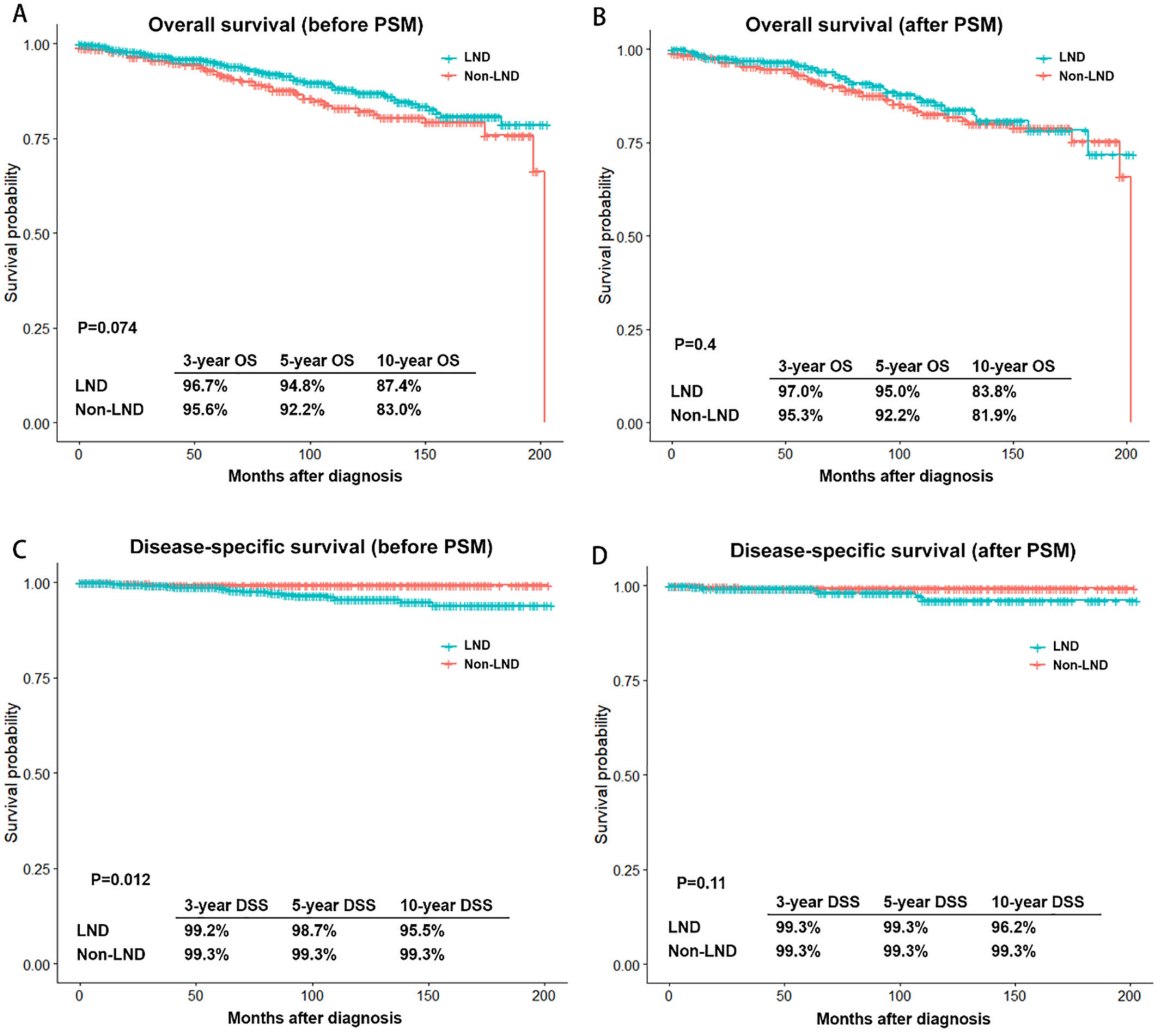
Outcomes and PSM-adjusted outcomes of patients with stage T1 medullary thyroid carcinoma between LND and non-LND groups. **(A)** Overall survival (OS) before PSM; **(B)** OS after PSM; **(C)** Disease-specific survival (DSS) before PSM; **(D)** DSS after PSM. PSM, Propensity score matching; LND, Lymph node dissection.

**Figure 3** reveals outcomes and PSM-adjusted outcomes between LND and non-LND groups. Results showed no significant advantage for LND over non-LND both before and after PSM. Additionally, the LND group and the non-LND group both achieved high 10-year disease-specific survival (DSS) rate, 96.2% and 99.3%, respectively.

### Subgroup analysis of lymph node dissection efficacy

**Figure 4:**
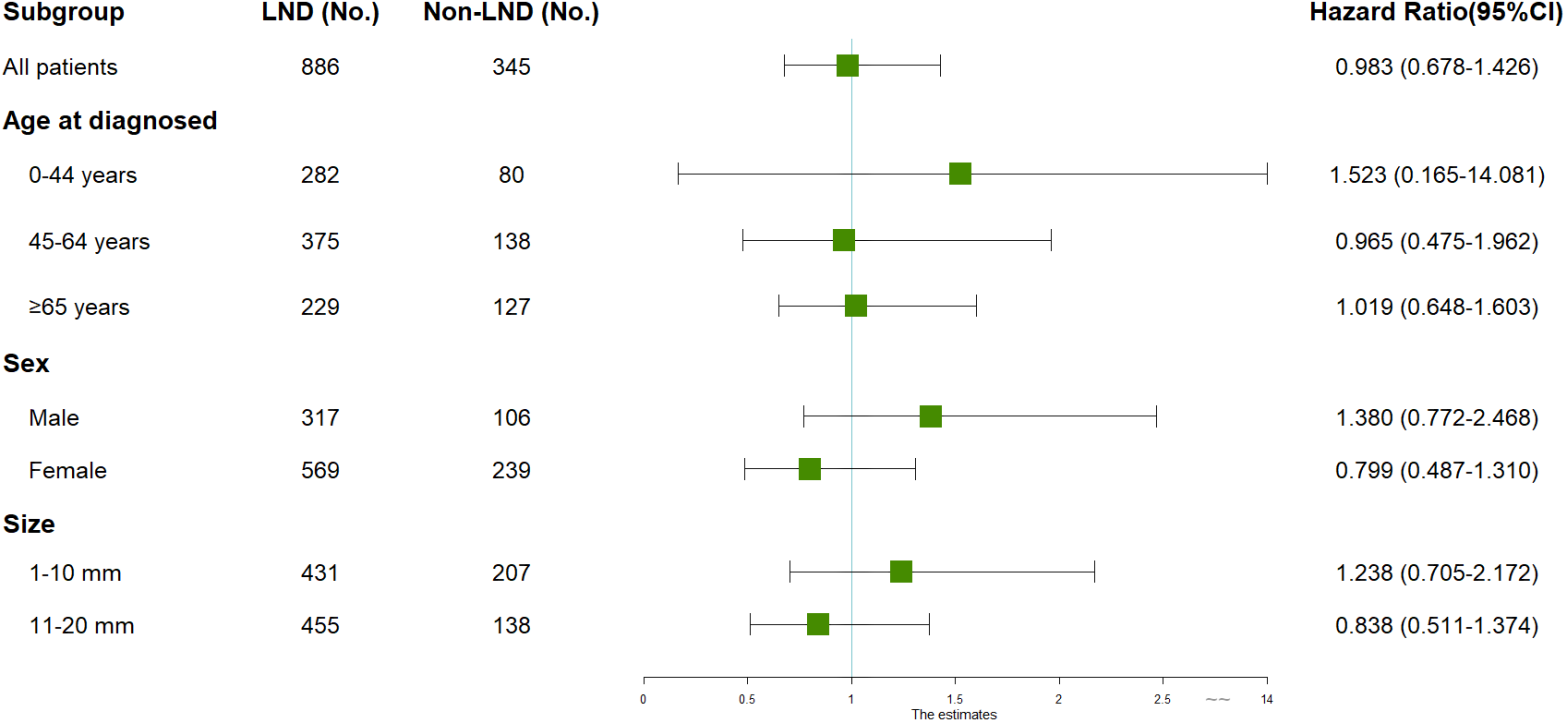
Subgroup analyses of patients with stage T1 medullary thyroid carcinoma by LND. All subgroup analyses were performed using multivariate cox regression analysis with overall survival as the outcome, adjusting for age, sex, marital status, race, and tumor size. CI, Confidence interval; LND, Lymph node dissection.

Although LND did not improve outcomes for all T1MTC patients, multivariable Cox regression analysis was employed to evaluate its potential efficacy in different subgroups. **Figure 4** demonstrates the results of the subgroup analysis. The results indicated no improved outcomes with LND in any subgroup, even in older age (HR=1.019, 0.648-1.603) and in patients with tumors size between 11-20 mm (HR=0.838, 0.511-1.374).

## Discussion

The standard surgical procedure, total thyroidectomy with bilateral central lymph node dissection with/without lateral lymph node dissection, recommended by ATA guidelines remains controversial for early-stage MTC patients (1). While several studies examined thyroidectomy extent, few evaluated optimal lymph node dissection (4–6, 13). Our findings showed T1MTC had a low lymph node metastasis rate (22%) and a relatively good prognosis (10-year DSS: 95.5-99.3%). Our results for the first time revealed that T1MTC patients who underwent total thyroidectomy with LND had no significant advantage over those underwent total thyroidectomy alone in both outcomes and PSM-adjusted outcomes. Further subgroup Cox analyses revealed no prognostic benefit of LND in any subgroup. Our study supported thyroidectomy without LND as a viable treatment option for T1MTC patients, providing an extra margin of safety for reducing the extent of lymph node dissection in T1MTC patients.

Recent epidemiological data showed an increased proportion of early-stage MTC patients in the United States, indicating that more attention should be paid to the management of these patients (15, 16). Our results revealed that T1MTCs were the main component of MTC patients, accounting for approximately 50.4% of cases. What’s more, our results showed these patients rarely died of MTC, with a good 10-year DSS rate (95.2-99.3%). Similarly, Gui et al. found the DSS of T1MTC patients with tumor size ≤10mm and those with tumor size >10mm was approximately 98% and 95%, respectively (17). Based on survival data and clinical practice, many surgeons view the standard procedure as overly aggressive for T1MTC patients, prompting studies on the surgical extent. Ito et al. followed 233 MTC patients for up to 433 months (median follow-up time 129 months) and found that unilateral thyroidectomy was an adequate local radical surgical procedure for patients with sporadic MTC (5). Similarly, Zhang et al. found no biochemical cure or OS benefit of total thyroidectomy over unilateral thyroidectomy in 129 unilateral sporadic MTC patients (6). In a pilot study, Dralle et al. proposed unilateral thyroidectomy with ipsilateral central LND as a curative strategy for non-desmoplastic sporadic MTC (13). However, these studies all focused on the extent of thyroidectomy, and the optimal scope of lymph node dissection for T1MTC patients has not been well-established (18).

One reason of lymph node dissection is necessary in MTC patients is the recognized high lymph node metastasis rate. However, the specific lymph node metastasis pattern in T1MTC patients has been poorly defined before (7, 18–21). In the last century, Moley et al. found that even early-stage patients had a high lymph node metastasis rate (>75%) (21). Due to the limitation of its time, this study included residual, recurrent, and delayed LND cases, potentially biasing results. Machens et al. conducted a retrospective cohort study and found that 26% of 233 medullary thyroid microcarcinoma patients experienced lymph node metastasis, and also provided literature review of lymph node metastasis rate in these patients (5%-31%) (22). To better establish lymph node metastasis patterns in MTC patients, we utilized the large sample cohort from the SEER database for the first time, finding the increased lymph node metastasis rate with higher T stage. Our population-based study showed that only 22.0% of T1MTC patients had lymph node metastasis, suggesting standard LND may be unnecessary in most T1MTC cases.

To validate our speculation and investigate the LND’s prognostic impact in T1MTC patients, we included T1MTC patients from the SEER database and divided them into LND and non-LND groups. The baseline differences demonstrated the higher elder patient rate and microcarcinoma rate in the non-LND group, indicating that these patients were more likely to undergo relatively conservative surgical procedures. However, the driving factors of this choice have not been well established, and may be related to the lower rate of lymph node metastasis in these patients (12, 22). Considering the potential impact of patients’ age and tumor size on the recurrence-free survival rate, overall survival rate, and disease-specific survival rate in MTC patients, we utilized PSM to reduce the baseline differences and achieved an approximate randomization effect (23–27). The consequent survival analyses revealed no outcome benefit with LND both before and after PSM, indicating that for T1MTC patients, LND failed to improve their prognosis. Furthermore, we tried to find some subgroups possibly beneficial from LND. However, multivariable Cox regression by different clinicopathological features showed that there was no subgroup beneficial from LND, even in subgroups with relatively worse prognosis, like elder patients and patients with tumor size >10mm. Therefore, we further validated that total thyroidectomy without LND is a viable treatment option for T1MTC patients, based on survival analyses and subgroup analyses. Since the incidence of T1MTC is still increasing, our findings may guide the treatment of more than half of MTC patients, improving their life quality with the similar prognosis to standard procedures.

However, it’s worth noting that, though our data suggested no overall LND benefit in T1MTC patients, individualized surgical procedures remained prudent. Since it is difficult to quantify the psychological stress caused by the abnormal postoperative calcitonin level resulting from the positive lymph node residual, LND was still a viable option in specific T1MTC patients. Preoperative examinations, like ultrasound and CT, can sensitively predict the presence of lymph node metastasis, and patients with positive preoperative examinations were recognized as cN1. For cN1 patients, LND in the positive region was a viable option. For cN0 cases, concerns exist regarding occult nodal metastases leading to biochemical persistence or recurrence (5, 18, 19, 28). For these concerns, Machens et al. investigated the lymph node metastasis pattern based on the basal calcitonin level, and found that tumor metastasized to ipsilateral central region first, then ipsilateral lateral region, then contralateral central region, contralateral lateral region and upper mediastinum region (28, 29). We also conducted Poisson regression and found that younger age, male, and tumor larger than 10mm were independent promotive factors for lymph node metastasis in T1MTC patients. So, based on the lymph node metastasis pattern and promotive factors for lymph node metastasis in MTC patients, there have been two surgical procedures to deal with occult lymph node metastasis, namely prophylactic LND and delayed LND. Prophylactic ipsilateral central lymph node dissection was an optional surgical procedure in partial cN0 patients who were worried about potential occult lymph node metastasis and might be overwhelmed by it, or in patients with several promotive features for lymph node metastasis (18, 19). For other cN0 patients, since delayed LND reduced the additional unnecessary surgery scope and complications, it was a relatively better procedure. Pena et al. proved that patients with delayed LND and those with prophylactic LND had a similar biochemical rate and survival rate (30). Kuo et al. found that reoperation didn’t increase the death rate and help achieve long-term local control (31). As per 2015 ATA guideline and past studies, postoperative serum calcitonin level was a sensitive and specific monitoring indicator for persistent or recurrent disease. The normalized postoperative serum calcitonin indicates the low risk of recurrence, while the high postoperative serum calcitonin means the potential persistent disease and need more imaging examinations, especially regular neck ultrasound (1, 32). Moreover, Xiao et al. provided an early-detection method of postoperative occult lymph node residual by the combination of ultrasound and calcitonin level to better guide the implementation of delayed LND (33).

In conclusion, our study supported thyroidectomy without LND as a viable treatment option for T1MTC patients, providing an extra margin of safety for reducing the extent of lymph node dissection in T1MTC patients. Surgeons can personalize the choice of standard LND, prophylactic LND, delayed LND, or non-LND based on the patient’s clinicopathological features, ultrasound findings, underlying condition, and expectations for quality of life. Our findings may help guide and revise the management of stage T1 medullary thyroid carcinoma.

There are several limitations to this study. Firstly, since the SEER database lacked genetic information and genetic testing was not widely implemented in clinical practice, we failed to conduct subgroup analysis by germline mutations. Though our results indicated that LND was unnecessary for T1MTC patients, the impact of LND on hereditary T1MTC patients was not established in our study. Further cohort studies with complete genetic information should be conducted to investigate the optimal LND scope in hereditary T1MTC patients. Secondly, due to the lack of specific indicators like serum calcitonin level and recurrence in the SEER database, our study focused on the distant prognosis (OS, DSS) and didn’t contain the short-term prognosis (biochemical cure, recurrence). Though distant prognosis represents final outcomes, further cohort studies with complete short-term prognosis were still needed for better completion of the conclusions, as the follow-up cost and psychological burden related to persistent structural diseases were difficult to quantization. Thirdly, to reduce the bias from incomplete surgery, our SEER cohort excluded patients who underwent unilateral thyroidectomy. Since several recent studies have investigated the possibility of unilateral thyroidectomy, further studies focusing on unilateral thyroidectomy with/without LND should be conducted to supplement our results.

## Conclusion

Conclusively, results from this population-based cohort study indicated that total thyroidectomy without lymph node dissection is a viable surgical procedure for stage T1 MTC patients, providing an extra margin of safety for narrowing the extent of lymph node dissection in nearly half of MTC patients. Further multi-central cohort studies should be conducted to verify our results.

## Data Availability

The original data presented in the study are available in SEER database. Further inquiries can be directed to the corresponding author.

## References

[1] Wells SA Jr. Asa SL Dralle H Elisei R Evans DB Gagel RF. Revised American Thyroid Association guidelines for the management of medullary thyroid carcinoma. Thyroid. (2015) 25:567–610. doi: 10.1089/thy.2014.0335 25810047 PMC4490627

[2] Haddad RI Bischoff L Ball D Bernet V Blomain E Busaidy NL. Thyroid carcinoma, version 2.2022, NCCN clinical practice guidelines in oncology. J Natl Compr Canc Netw. (2022) 20:925–51. doi: 10.6004/jnccn.2022.0040 35948029

[3] Filetti S Durante C Hartl D Leboulleux S Locati LD Newbold K. Thyroid cancer: ESMO Clinical Practice Guidelines for diagnosis, treatment and follow-up†. Ann Oncol. (2019) 30:1856–83. doi: 10.1093/annonc/mdz400 31549998

[4] Ito Y Miyauchi A Yabuta T Fukushima M Inoue H Tomoda C. Alternative surgical strategies and favorable outcomes in patients with medullary thyroid carcinoma in Japan: experience of a single institution. World J Surg. (2009) 33:58–66. doi: 10.1007/s00268-008-9795-2 19005720

[5] Ito Y Miyauchi A Kihara M Higashiiyama T Fukushima M Miya A. Static prognostic factors and appropriate surgical designs for patients with medullary thyroid carcinoma: the second report from a single-institution study in Japan. World J Surg. (2018) 42:3954–66. doi: 10.1007/s00268-018-4738-z PMC6244981 30051240

[6] Zhang J Gu P Huang D Zhao J Zheng X Gao M. Surgical selection and prognostic analysis in patients with unilateral sporadic medullary thyroid carcinoma. Langenbecks Arch Surg. (2022) 407:3013–23. doi: 10.1007/s00423-022-02591-9 35748956

[7] Weber T Schilling T Frank-Raue K Colombo-Benkmann M Hinz U Ziegler R. Impact of modified radical neck dissection on biochemical cure in medullary thyroid carcinomas. Surgery. (2001) 130:1044–9. doi: 10.1067/msy.2001.118380a 11742336

[8] Spanheimer PM Ganly I Chou JF Capanu M Nigam A Ghossein RA. Prophylactic lateral neck dissection for medullary thyroid carcinoma is not associated with improved survival. Ann Surg Oncol. (2021) 28:6572–79. doi: 10.1245/s10434-021-09683-8 PMC8452790 33748897

[9] van Beek DJ Almquist M Bergenfelz AO Musholt TJ Nordenstrom E Council E. Complications after medullary thyroid carcinoma surgery: multicentre study of the SQRTPA and EUROCRINE(R) databases. Br J Surg. (2021) 108:691–701. doi: 10.1093/bjs/znaa195 34157081 PMC10364906

[10] Park SY Cho YY Kim HI Choe JH Kim JH Kim JS. Clinical validation of the prognostic stage groups of the eighth-edition TNM staging for medullary thyroid carcinoma. J Clin Endocrinol Metab. (2018) 103:4609–16. doi: 10.1210/jc.2018-01386 30137493

[11] Bae SY Jung SP Choe JH Kim JS Kim JH. Prediction of lateral neck lymph node metastasis according to preoperative calcitonin level and tumor size for medullary thyroid carcinoma. Kaohsiung J Med Sci. (2019) 35:772–77. doi: 10.1002/kjm2.12122 PMC11900780 31483088

[12] Chandeze M-M Noullet S Faron M Trésallet C Godiris-Petit G Tissier F. Can we predict the lateral compartment lymph node involvement in RET-negative patients with medullary thyroid carcinoma? Ann Surg Oncol. (2016) 23:3653–59. doi: 10.1245/s10434-016-5292-2 27271930

[13] Dralle H Brandenburg T Weber F Führer-Sakel D Theurer S Baba HA. Sporadic noninvasive medullary thyroid neoplasm: A desmoplasia-negative unifocal nonmetastatic tumor cured by hemithyroidectomy. Surgery. (2023) 174:1356–62. doi: 10.1016/j.surg.2023.09.003 37821265

[14] von Elm E Altman DG Egger M Pocock SJ Gøtzsche PC Vandenbroucke JP. The Strengthening the Reporting of Observational Studies in Epidemiology (STROBE) statement: guidelines for reporting observational studies. J Clin Epidemiol. (2008) 61:344–9. doi: 10.1016/j.jclinepi.2007.11.008 18313558

[15] Tao Z Deng X Guo B Ding Z Fan YB. Subgroup analysis of steadily increased trends in medullary thyroid carcinoma incidence and mortality in the United States, 2000-2020: a population-based retrospective cohort study. Endocrine-related Cancer. (2024) 31(5):e230319. doi: 10.1530/erc-23-0319 38376827 PMC11046345

[16] Randle RW Balentine CJ Leverson GE Havlena JA Sippel RS Schneider DF. Trends in the presentation, treatment, and survival of patients with medullary thyroid cancer over the past 30 years. Surgery. (2017) 161:137–46. doi: 10.1016/j.surg.2016.04.053 PMC5164945 27842913

[17] Gui Z Wang Z Xiang J Sun W He L Dong W. Incidental T1 stage medullary thyroid carcinoma: The effect of tumour diameter on prognosis and therapeutic implications. Clin Endocrinol. (2022) 97:355–62. doi: 10.1111/cen.14702 35192214

[18] Fussey JM Bradley PJ Smith JA. Controversies in the surgical management of sporadic medullary thyroid carcinoma. Curr Opin Otolaryngol Head Neck Surg. (2020) 28:68–73. doi: 10.1097/moo.0000000000000612 32011397

[19] Polistena A Sanguinetti A Lucchini R Galasse S Monacelli M Avenia S. Timing and extension of lymphadenectomy in medullary thyroid carcinoma: A case series from a single institution. Int J Surg. (2017) 41 Suppl 1:S70–4. doi: 10.1016/j.ijsu.2017.04.026 28506418

[20] Das S Jha CK Singh PK. Extent of surgery in localized, sporadic medullary thyroid carcinoma: Can we avoid central compartment lymph node dissection? Surgery. (2023) 174:1094–95. doi: 10.1016/j.surg.2022.12.015 36639300

[21] Moley JF DeBenedetti MK. Patterns of nodal metastases in palpable medullary thyroid carcinoma: recommendations for extent of node dissection. Ann Surg. (1999) 229:880–7;discussion 87-8. doi: 10.1097/00000658-199906000-00016 10363903 PMC1420836

[22] Machens A Dralle H. Biological relevance of medullary thyroid microcarcinoma. J Clin Endocrinol Metab. (2012) 97:1547–53. doi: 10.1210/jc.2011-2534 22399508

[23] Rubin DB. Estimating causal effects from large data sets using propensity scores. Ann Internal Med. (1997) 127:757–63. doi: 10.7326/0003-4819-127-8_part_2-199710151-00064 9382394

[24] Opsahl EM Akslen LA Schlichting E Aas T Brauckhoff K Hagen AI. Trends in diagnostics, surgical treatment, and prognostic factors for outcomes in medullary thyroid carcinoma in Norway: A nationwide population-based study. Eur Thyroid J. (2019) 8:31–40. doi: 10.1159/000493977 30800639 PMC6381913

[25] Sahli ZT Canner JK Zeiger MA Mathur A. Association between age and disease specific mortality in medullary thyroid cancer. Am J Surg. (2021) 221:478–84. doi: 10.1016/j.amjsurg.2020.09.025 PMC9169693 33010878

[26] Gogna S Goldberg M Samson D Gachabayov M Felsenreich DM Azim A. Medullary thyroid cancer in patients older than 45-epidemiologic trends and predictors of survival. Cancers (Basel). (2020) 12(11):3124. doi: 10.3390/cancers12113124 33114488 PMC7692716

[27] Wu X Li B Zheng C. Clinicopathological characteristics and prognosis of medullary thyroid microcarcinoma: a tumor with a similar prognosis to macrocarcinoma. Eur J Med Res. (2023) 28:546. doi: 10.1186/s40001-023-01534-4 38017592 PMC10683302

[28] Machens A Hauptmann S Dralle H. Prediction of lateral lymph node metastases in medullary thyroid cancer. Br J Surg. (2008) 95:586–91. doi: 10.1002/bjs.6075 18300267

[29] Machens A Dralle H. Biomarker-based risk stratification for previously untreated medullary thyroid cancer. J Clin Endocrinol Metab. (2010) 95:2655–63. doi: 10.1210/jc.2009-2368 20339026

[30] Pena I Clayman GL Grubbs EG Bergeron JM Jr Waguespack SG Cabanillas ME. Management of the lateral neck compartment in patients with sporadic medullary thyroid cancer. Head Neck. (2017) 40:79–85. doi: 10.1002/hed.24969 29044788

[31] Kuo EJ Sho S Li N Zanocco KA Yeh MW Livhits MJ. Risk factors associated with reoperation and disease-specific mortality in patients with medullary thyroid carcinoma. JAMA Surg. (2018) 153(1):52–9. doi: 10.1001/jamasurg.2017.3555 28973144 PMC5833622

[32] Prete A Gambale C Torregrossa L Ciampi R Romei C Ramone T. Clinical evolution of sporadic medullary thyroid carcinoma with biochemical incomplete response after initial treatment. J Clin Endocrinol Metab. (2023) 108:e613–e22. doi: 10.1210/clinem/dgad061 36722192

[33] Xiao J Jiang J Chen W Hong T Li B He X. Combination of ultrasound and serological tests for detecting occult lateral lymph node metastases in medullary thyroid cancer. Cancer Med. (2023) 12:11417–26. doi: 10.1002/cam4.5856 PMC10242362 37004158

